# Unveiling the radiation shielding efficacy of diorite, granodiorite, tonalite, and granite: experimental and simulation study

**DOI:** 10.1038/s41598-024-82081-8

**Published:** 2025-01-04

**Authors:** M. Elsafi, M. A. El-Nahal, M. K. Alawy, Islam M. Nabil

**Affiliations:** 1 Physics Department, Faculty of Science, Alexandria University, Alexandria, 21511 Egypt; 2 Department of Environmental Studies, Institute of Graduate Studies and Research, Alexandria University, Alexandria, 21511 Egypt; 3 MEU Research Unit, Middle East University, Amman, Jordan; 4 Geology Department, Faculty of Science, Alexandria University, Alexandria, 21511 Egypt; 5 Physics Department, Faculty of Science, Fayoum University, Fayoum, Egypt

**Keywords:** Rocks samples, Granodiorite, Tonalite, Diorite, Granite, Radiation shielding, MCNP5, Techniques and instrumentation, Materials science, Physics

## Abstract

For the purpose of this study, four natural rock samples-namely, diorite, granodiorite, tonalite, and granite-are being investigated about their radiation attenuation. The elemental composition of the rocks was obtained through Energy dispersive X-ray spectroscopy (EDX) which examines the microstructural and localized area elemental analyses of the four rock samples. A Monte Carlo simulation (MCNP) was used to determine and evaluate the investigated samples. Additionally, the samples were validated by Phy-X software (within the energy range of 0.015 to 15 MeV), and experimental measurements were achieved through the utilization of an HPGe detector (0.060, 0.662, 1.173, and 1.332 MeV). The investigation was carried out using various parameters such as linear attenuation (µ) and others. Furthermore, the Fast Neutrons Removal Cross Sections (FNRCS) were calculated using theoretical methods. In the case of granodiorite, tonalite, diorite, and granite, the values of µ were found to range from 7.931 to 0.049, 31.922 to 0.061, 17.267 to 0.060, and 23.860 to 0.056 cm^−1^, respectively. The samples of tonalite and diorite have the highest µ values due to the presence of heavy elements and the high densities of these samples. Granodiorite is the substance that possesses the highest value of FCS (0.108 cm^−1^) due to the high content of light elements (O = 0.6802%, and C = 0.2286% wt). The results of the study demonstrated that the investigated natural rocks possessed a substantial potential for shielding γ-rays and neutrons from radiation and could be suitable for use in radiological protection applications.

## Introduction

Devices that generate artificial ionizing radiation, such as X-rays and γ-rays, have been widely adopted in a variety of industrial, medical, and nuclear configurations. This widespread adoption is a direct result of the utilization of technological breakthroughs^1,2^. Nevertheless, prolonged and excessive exposure to such radiation can have adverse effects on health, potentially leading to the development of cancer, as well as symptoms such as vomiting, nausea, and, in severe situations, even mortality^3^. The interaction of high-energy photons with human tissue results in the ionization of water molecules within the tissue. Due to the fact that it causes harm to both the exterior surface and the inside content of DNA, this ionization should be avoided at all costs. Neutrons, γ-rays, and X-rays pose a threat to the environment, as well as to people and animals. For all of these reasons, the pursuit of discovering improved materials for radiation attenuation and shielding is something that a lot of researchers are interested in Refs^4–8^. To decrease the amount of hazardous radiation that workers are exposed to, it is well known that shielding and attenuation materials act as a barrier between the sources of radiation that release radiation and the surrounding area or the workers themselves. When it comes to minimizing or reducing the potentially harmful effects of radiation, one of the fundamental concepts of radiation protection is the selection of appropriate shielding materials. This is one part of the radiation protection process. This particular selection is of utmost significance. This specific aspect of the situation is still the subject of investigations and study inquiries that are currently being carried out. Polymers, natural rocks, rubber, concrete, brick, and alloy are just some of the materials that have been investigated in a number of studies that have been published in the literature. Other materials that have been investigated include polymers^9,10^. These studies have been conducted to investigate the effectiveness of these materials as radiation shields.

For the purpose of conducting research on the radiation attenuation properties of these rocks, a collection of naturally occurring rocks has been selected from the Central Eastern Desert of Egypt. This collection will be used for the purpose of conducting the research. Examples of intrusive igneous rocks that are included in the selected group include diorite, granodiorite, tonalite, and granite. These are just a few examples. There is a gradual cooling of magma, molten rock, that takes place underground, ultimately leading to the formation of these rocks. The amount of silica in these rocks is somewhat moderate, and the amount of alkali metals they contain is relatively low^11,12^. Since these natural materials have been used as building materials since the beginning of human civilization, it is essential to study their attenuation properties. These rocks are characterized by their heavy density with an average of 2.75 g cm^−3^, hardness, and durability^12^, so they are very convenient to use as radiation protection building materials. Moreover, various types of high-resistance anti-radiation concrete can be synthesized by using granites or granite-like rock waste as fine or coarse aggregates of anti-concrete mix^13^. The melting point of igneous rocks is high enough to bear the thermal effect of being exposed to a high dose of ionizing radiation, which ranges from 1215 to 1260 °C at ambient pressure. Contrary to marbles, granites or granite-like rock have good resistance to weathering processes^14^, and the average porosity of granites is about 1% (very low), so its hydraulic conductivity is very low also and equals about 0.0041 m/day^15^. Therefore, the physical properties of the materials that were investigated are very suitable for operation in harsh radiological environments, such as those found in nuclear reactors. Furthermore, these igneous rocks that are readily available in the environment have the potential to be effective radiation-shielding material candidates. They also represent an environmentally friendly and convenient alternative to conventional radiation-shielding materials such as lead, tungsten, and steel, as well as the extraction, mining, and manufacturing processes that have significant negative effects on the environment and human health^16–18^.

Using experimental and theoretical methods, Kadir Günoğlu et al. examines the γ-radiation shielding properties of igneous rocks from Isparta, Turkey. LAC was examined in relation to rock density and alkali-silica content. LACs increased with rock and alkali-silica ratio. XCOM software was used to theoretically calculate LACs from the chemical content of selected igneous rocks. According to the data, the trachy-basalt sample from the igneous rocks used in this study shields γ-radiation better than other rocks^19^. Masoud et al. evaluates the attenuation efficiency of Egyptian barite and hematite minerals against fast neutrons and γ-rays in their natural state. A Pu-Be source and stilbene scintillator were used to measure radiation attenuation against fast neutrons and γ-rays. NXcom was used to validate fast neutron attenuation measurements with theoretical calculations. Barite outperforms hematite in radiation attenuation efficiency for fast neutrons and γ-rays by 9.17 and 51%, respectively. The experimental and theoretical results agreed well, with barite and hematite samples deviating by 16 and 19.25%, respectively. Barite and hematite may become sustainable RSC alternatives^20^. M. A. Rashwan et al. described the physico-mechanical properties and shielding efficiency of the Neoproterozoic Um Had composite granitoid pluton using mineralogical and geochemical compositions. This study investigates the gamma radiation shielding potential of the syenogranite samples. The research indicates an inverse relationship between photon energy and G_MAC_, with the highest values in the (I) granite sample (∼18). This study shows our samples’ promising radiation shielding. The findings of G_MAC_, Z_eff_, EBF, and EABF can help develop natural radiation shielding materials^21^.

By measuring the linear/mass attenuation factors theoretically and experimentally, this study aims to determine the shielding properties against radiation of the selected types of rocks (diorite, granodiorite, tonalite, and granite). Following this, the attenuation parameters, such as the half value layer and radiation protection efficiency, will be deduced. This will allow for the evaluation of the effectiveness of using these materials as radiation protection materials and the determination of which radiation shielding applications these materials can be used for.

## Materials and methodology

### Materials

**Fig. 1:**
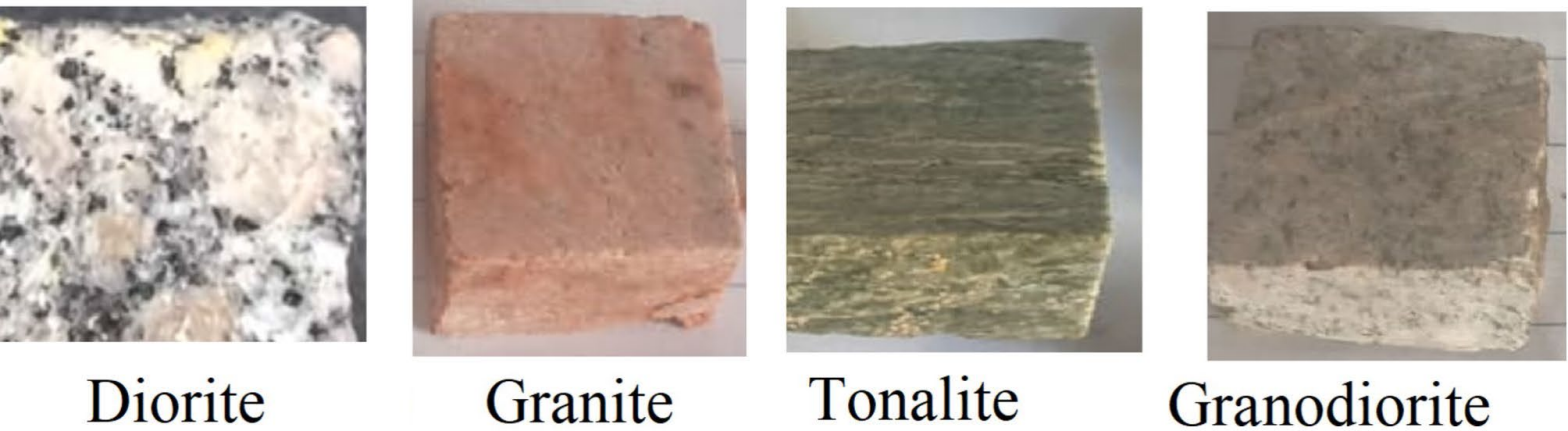
Samples images for investigated rock samples.

Four samples have been selected from the Central Eastern desert of Egypt (Fig. 1) which are diorite, granodiorite, tonalite, and granite. These rocks are igneous rocks which crystallize and solidify from magma in deep levels from the earth crust. So, they are very hard, massive, and have high specific gravity. Depending on this nature, they have variable uses such as crushed stones for road building, construction materials, paving, countertops, tile floors, in addition they were used in construction of ancient pharaonic statues. The investigated rocks are part of the basement rocks of Egypt. These rocks form the Red Sea Mountains, which cover a vast area of Egyptian Eastern Desert and stretch roughly 800 km southward to Northern Sudan. In addition, they exist in the southern sector of Sinai, the southwestern part of the western desert, and at Aswan around the Nile valley. Diorite occurs as several outcrops of small extension. Granodiorite and tonalite represent 27% of basement rocks, whereas granite constitutes 30%. Each of these rocks has a significant economic importance. They serve a variety of domestic purposes. For instance, they are used in construction materials, road paving, railway tracks construction, building statues, and ornamental stone. Polished granite and granodiorite pieces are utilized indoors for countertops, stair treads, and tile floors. In addition, gold-bearing quartz veins are found mainly in granodiorite, tonalite, and granite which are the host rocks for most gold mineralization activities in Egypt. Diorite is characterized by mix of black and white minerals. It consists of amphiboles, plagioclase, and minor pyroxene. Granodiorite is light grey in color and composes of quartz, alkali-feldspars, hornblende, plagioclase feldspar, and biotite. Tonalite is similar to granodiorite but differs in the modal content of the plagioclase feldspar which is more abundant (up to 40% of the rock mode) in tonalite. Granite is red to reddish brown color due to presence of high percent of orthoclase. It consists of orthoclase, quartz, microcline, plagioclase, and biotite. The four rock types have accessory minerals such as zircon, titanite, and apatite. The investigated rocks, with typical chemical compositions shown in Table 1, were handled in the form of 3 mm in thickness. They were cut in the form of 70 × 70 mm squares to fit the supporting frame in front of the experimental work.

**Table 1:** Chemical composition, and density of the investigated rocks samples.

Sample	Composition wt.%									Density (g cm^− 3^)
	CO_2_	Na_2_O	MgO	Al_2_O_3_	SiO_2_	K_2_O	CaO	TiO_2_	FeO	
Granodiorite	0.838	0.002	–	0.014	0.092	0.004	0.050	–	–	2.600
Tonalite	–	0.049	0.025	0.207	0.490	0.023	0.082	0.018	0.106	2.790
Diorite	–	–	0.006	0.042	0.930	0.012	0.011	–	–	2.870
Granite	0.281	0.014	0.045	0.126	0.380	0.013	0.044	–	0.097	2.660

### Methodology

Energy dispersive X-ray spectroscopy (EDX) divides the following investigation into two main parts. The first section examines the microstructural and localized area elemental analyses of the four rock samples. In part two of the review, a theoretical assessment of both γ-rays and fast neutrons is presented, and an experimental investigation of γ-ray attenuation is also included. Reliable software tools, which will be described in detail in the following sections, were used to conduct this evaluation. The EDX works in conjunction with a scanning electron microscope, where the sample is carefully prepared, often by cutting it into small pieces or grinding it to prepare it for analysis, and more than one area of the sample is analyzed. Electron microscopes are used to shine a beam of electrons on the sample. This beam causes electrons to be emitted from the elements in the sample. The energy from the emitted electrons is collected in the EDX system. Each element has a specific energy for the emitted electrons, allowing the elements in the sample to be identified. EDX produces a spectrum that shows peaks representing the different elements as shown in Fig. 1. These peaks are analyzed to determine the active elements and their amounts.

#### Radiation shielding investigation

##### Theoretical basis

We need the variables’ shielding parameters of interest (e.g., µ, µ_c_, etc.)^22,23^, the appropriate mathematical equations and definitions, before we can show you the experimental and theoretical methods used in this study and explain what we hope to accomplish with this sections^24–30^:

1 $${\text{I}}\,=\,{{\text{I}}_{\text{o}}}{{\text{e}}^{ - {\text{mx}}}},$$

2 $${^{\mu }_{\text{c}}}=\frac{\mu }{\rho },$$

3 $$HV\:=\:\frac{\text{l}\text{n}2}{{\upmu\:}}\:,\:\:\:$$

4 $$\:\:\text{T}\text{V}=3.32{\text{H}}_{1/2},\:\:$$

5 $$\:\text{M}\text{F}=\:\frac{1}{{\upmu\:}}\:\:.\:\:$$

The radiation protection (RP) is a crucial metric to consider when assessing the extent to which different shielding materials can reduce radiation. The transfer factor (TF) measures the extent to which photons are passed through the rock samples. The calculation of the RP and TF factors is as follows^31–36^:

6 $$\:RP,{\%}=\left(1-\frac{I}{{I}_{o}}\right)100,$$

7 $$\:\text{T}\text{F},{\%}\:=\:\left(\frac{I}{{I}_{o}}\right)100.$$

**Fig. 2:**
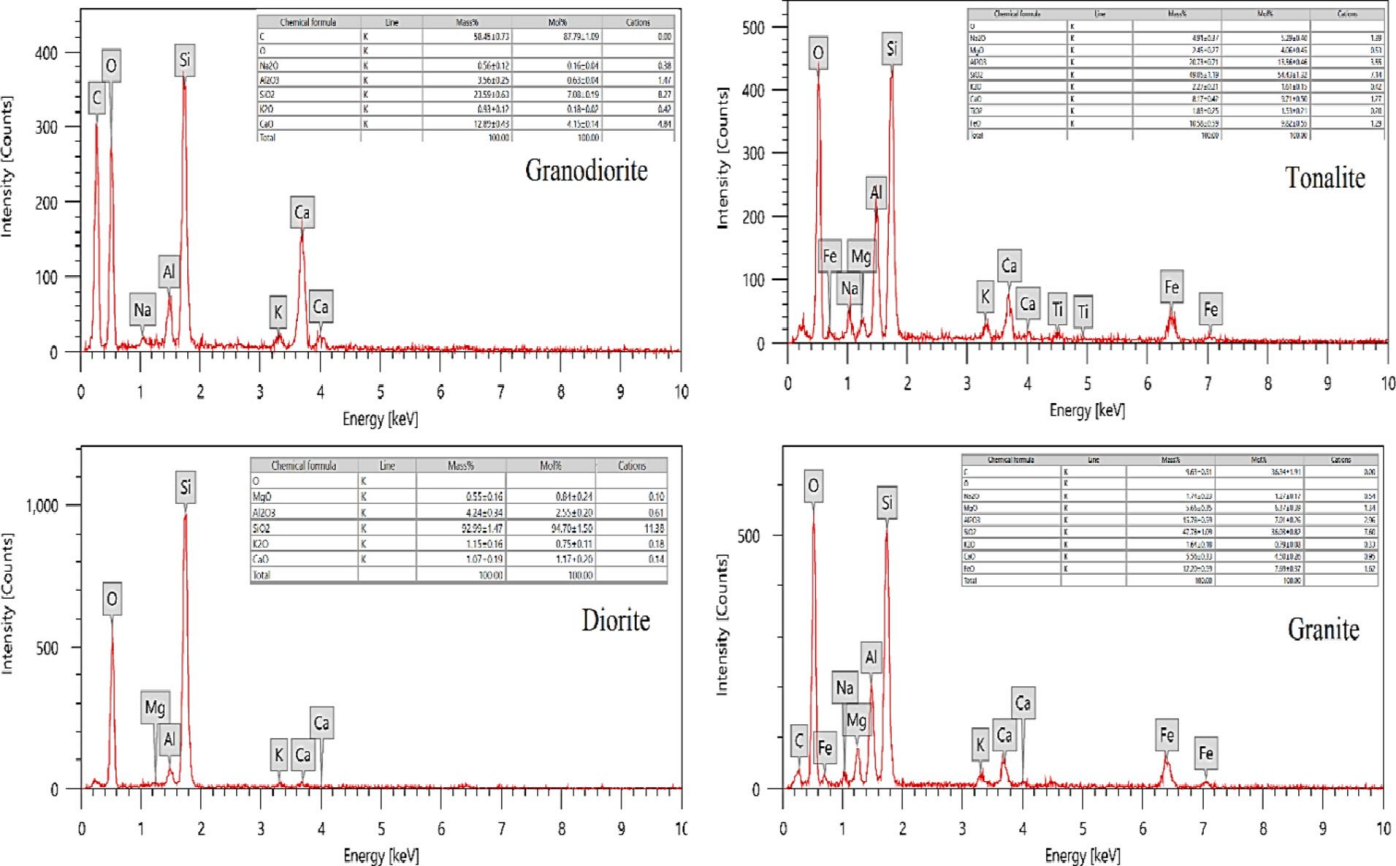
EDX-analysis of present rocks.

I denote the magnitude of the γ-rays that have penetrated the material. The µ_c_, the half-value (H_1/2_), the tenth value (T_1/10_), the mean free path (MF), and the intensity of the main gamma without the material are all represented by Io. In contrast, x represents the thickness of the material (Fig. 2).

The neutron attenuation potential of suggested materials may be determined by calculating the fast neutron removal cross-section (FCS) using the equation FCS =$\:\sum\:_{i}{W}_{i}$ x ρ. The symbol $\:{W}_{i}\:$represents the partial density, ρ represents the material density, and the subscript i represents the mass cross-section (σ) of the component)^37,38^. The half-value layer (HVL_FCS_) was determined using the formula. $\:{HVL}_{\text{F}\text{C}\text{S}}\:=\:\frac{ln2}{FCS}$, and the relaxation length (λ_FCS_) was calculated as $\:\frac{1}{FCS}$^39,40^.

##### Experimental measurements

**Fig. 3:**
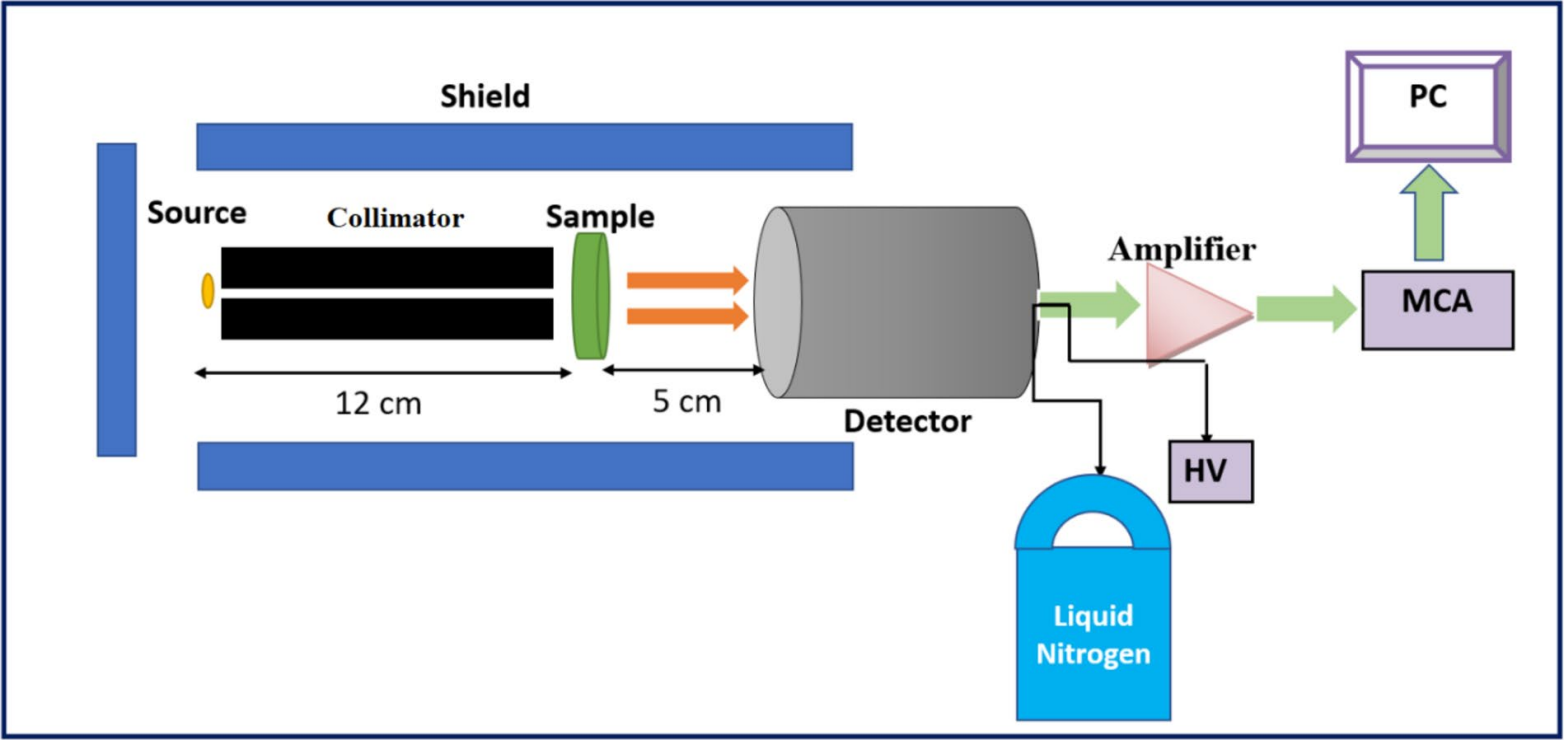
Experimental setup.

A preliminary experimental investigation was carried out using three different γ-ray sources, namely Am-241, Cs-137, and Co-60. The purpose of this study was to evaluate the existing state of affairs and investigate the degree of agreement between the γ-ray shielding parameters that were measured empirically and those that were anticipated theoretically. It wasn’t until after this that the opportunity to proceed with the theoretical inquiry was considered acceptable. Using the radioactive sources that were described earlier, the experimental γ-ray shielding efficiency of the rock samples that were being investigated was measured at four distinct energies: 0.060, 0.662, 1.173, and 1.332 MeV. For the purpose of carrying out the experimental testing, an HPGe detector was utilized in conjunction with a multichannel analyzer that was operating software (Genni-2000)^41^. A 3.5 cm internal cylindrical lead holder with a 3 mm aperture was used to house the γ-rays radioactive source. This holder was surrounded by an externally hollowed 10 cm lead cylinder, which was the source collimator. Additionally, the detector was shielded by a cylindrical lead shield, which served as the detector collimator. This was done to protect against scattered γ-rays and background radiation, as well as to maximize the accuracy of the readings^42^. The rock samples were stacked between the abovementioned collimators, and a straight vertical alignment for all components of the experimental setup was used. All of the particulars of the experimental setup, which are depicted in Fig. 3, are primarily taken into consideration in order to guarantee a narrow beam geometry and significantly reduce the build-up factors, which ultimately results in the acquisition of precise characteristic shielding parameters for the rock samples that are being investigated^43,44^.

##### Theoretical and simulation work

**Fig. 4:**
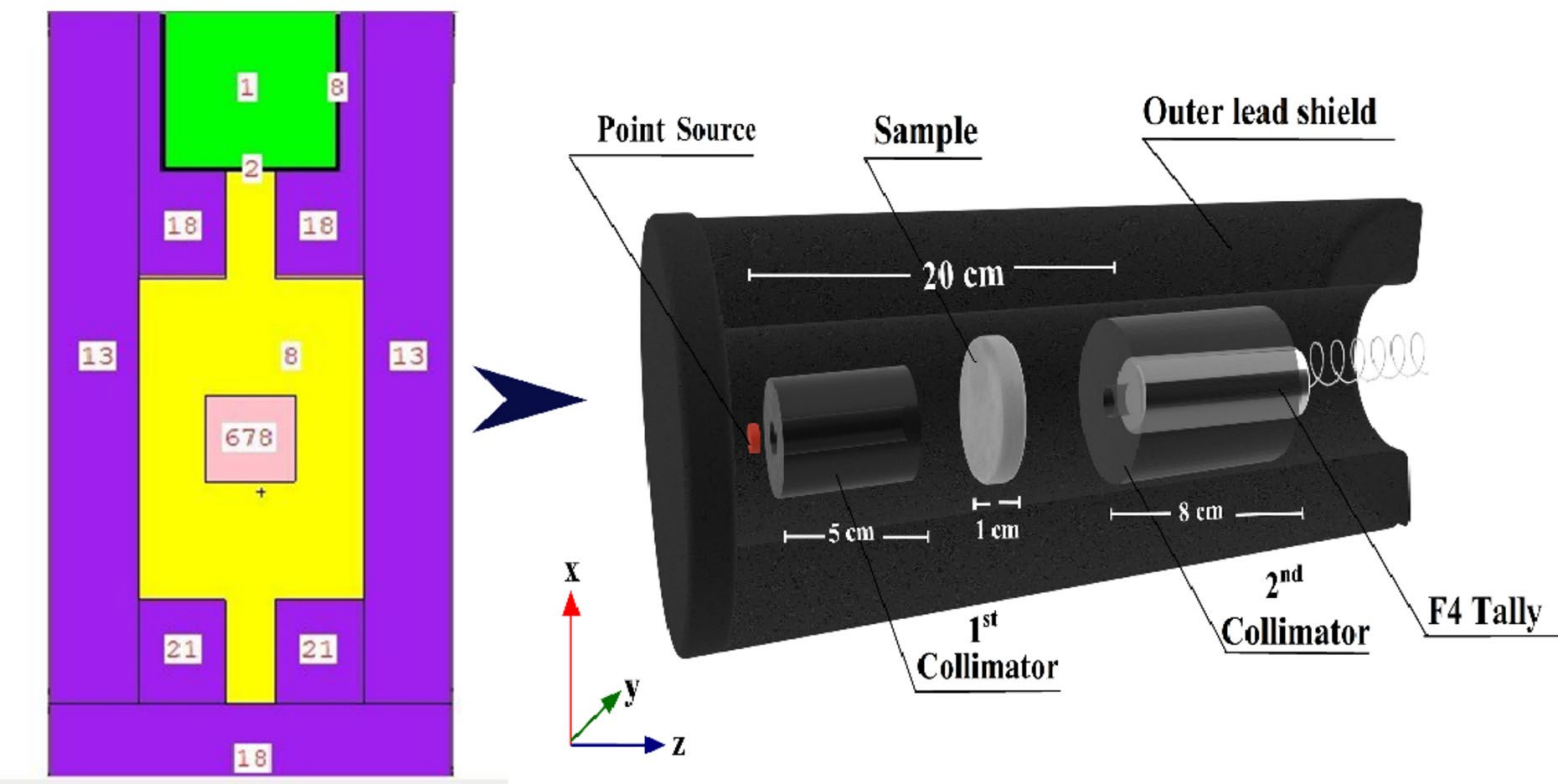
3-D dynamic view of the radiation attenuation simulation system used for the investigated rock samples.

Using the Monte Carlo technique, the code is built to mimic real-world particles. In order to predict the theoretical strength of γ-rays released by γ-point sources, the MCS simulation system was employed. An energetic γ-emitter source operating in the photon energy range (Pγ) of 0.015 ≤ Pγ ≤ 15 in MeV was included in the simulation^34,35^. A comparison of the γ-ray intensity before and after passing through the investigated glass materials was the intended objective. Radiation safety and shielding, dose calculation, detector design, and other research fields frequently favor MCS codes^45–47^. Reasons for this preference include these codes’ many useful features, such as fast calculations, adaptability to different geometrical designs, and operation across a wide range of energies. This method takes into account various photon interaction mechanisms and attempts to make electrons, neutrons, and γ-rays more mobile. Running an MCS simulation requires precise data on the geometry, source-to-detector distance, source dimensions, and chemical and elemental composition and densities of the concrete samples being examined. This data is found on the SDEF card^48,49^. A predetermined two-dimensional and three-dimensional setup served as the basis for the development of the simulation’s geometric configuration, which can be seen in Fig. 4. In accordance with the experimental system, each and every parameter has been recognized as being consistent. Text lines were used to generate the input files so that the MCS simulation could be run. There were six distinct components that comprised the cell, which included a radioactive source, a collimator for 1rays and 2rays γ-radiations, a sample with a cubic shape, and a detector. An SDEF mono γ-energetic flow was identified as a point source of γ-rays for every input file that fell within the range of 0.015 ≤ Pγ ≤ 15 MeV during the analysis. It has been established that the neutron source is a californium spectrum, which functions within the En ≤ 11 MeV range for the purpose of achieving rapid elimination σ attenuation. The specimens emerged as a cubic layer during the generation process. In addition to this, the densities and elemental compositions of the specimens that were examined were documented in the material card of the text lines. Within a lead collimator that was specifically designed for the 2nd γ-rays, the detector was effectively mounted. By using a Tally command, we may add up all the values from F4:P. At the same time, the F4:N method calculates the average duration of the γ-rays and neutrons emitted by simulated γ/neutron radiation sources. To protect the generated detector, collimators, source, and specimens under investigation, a lead outer shield was used. An Intel Core i5 was utilized to carry out the calculations. Multiple NPS (11^7^) attempts were carried out for every single file to guarantee that the random statistical errors remained below 1%. Also, the PhyX software was used to validate the experimental measurements and the simulation results.

## Results and discussion

### γ-rays shielding experimental assessment

According to the experimental setup and methodology described above, the transmission curves have been compiled at the abovementioned four γ-rays’ energies for the studied rock samples. The measurements have been performed as triplets, and the absolute value of the slope is recorded as the experimentally obtained linear attenuation (µ) value of the alloy under study. Table 2 presents the experimental and corresponding PhyX computed µ (cm^−1^) values at the four studied γ-rays’ energies for the investigated rock samples^50^. There was a good agreement between the experimental measurements and the values obtained from the Phy-x software with a maximum relative difference 4.78%.

**Table 2:** Experimental values at different discussed energies compared with Phy-x results.

Energy (MeV)	Gamma source	Granodiorite			Tonalite		
		PhyX	Exp	Dev %	PhyX	Exp	Dev %
0.060	*Am (241)*	0.5532	0.5332	3.76	1.0076	0.9690	3.98
0.662	*Cs (137)*	0.2008	0.1938	3.62	0.2134	0.2089	2.19
1.173	*Co (60)*	0.1529	0.1501	1.89	0.1622	0.1593	1.83
1.333	*Co (60)*	0.1432	0.1403	2.11	0.1519	0.1478	2.78

Energy (MeV)	Gamma source	Diorite			Granite		
		PhyX	Exp	Dev %	PhyX	Exp	Dev %
0.060	*Am (241)*	0.7430	0.7091	4.78	0.8504	0.8356	1.77
0.662	*Cs (137)*	0.222	0.2164	2.41	0.204	0.1993	2.44
1.173	*Co (60)*	0.169	0.1660	1.53	0.155	0.1504	3.22
1.333	*Co (60)*	0.158	0.1562	1.11	0.145	0.1426	1.98

### γ-rays shielding analytical assessment

**Fig. 5:**
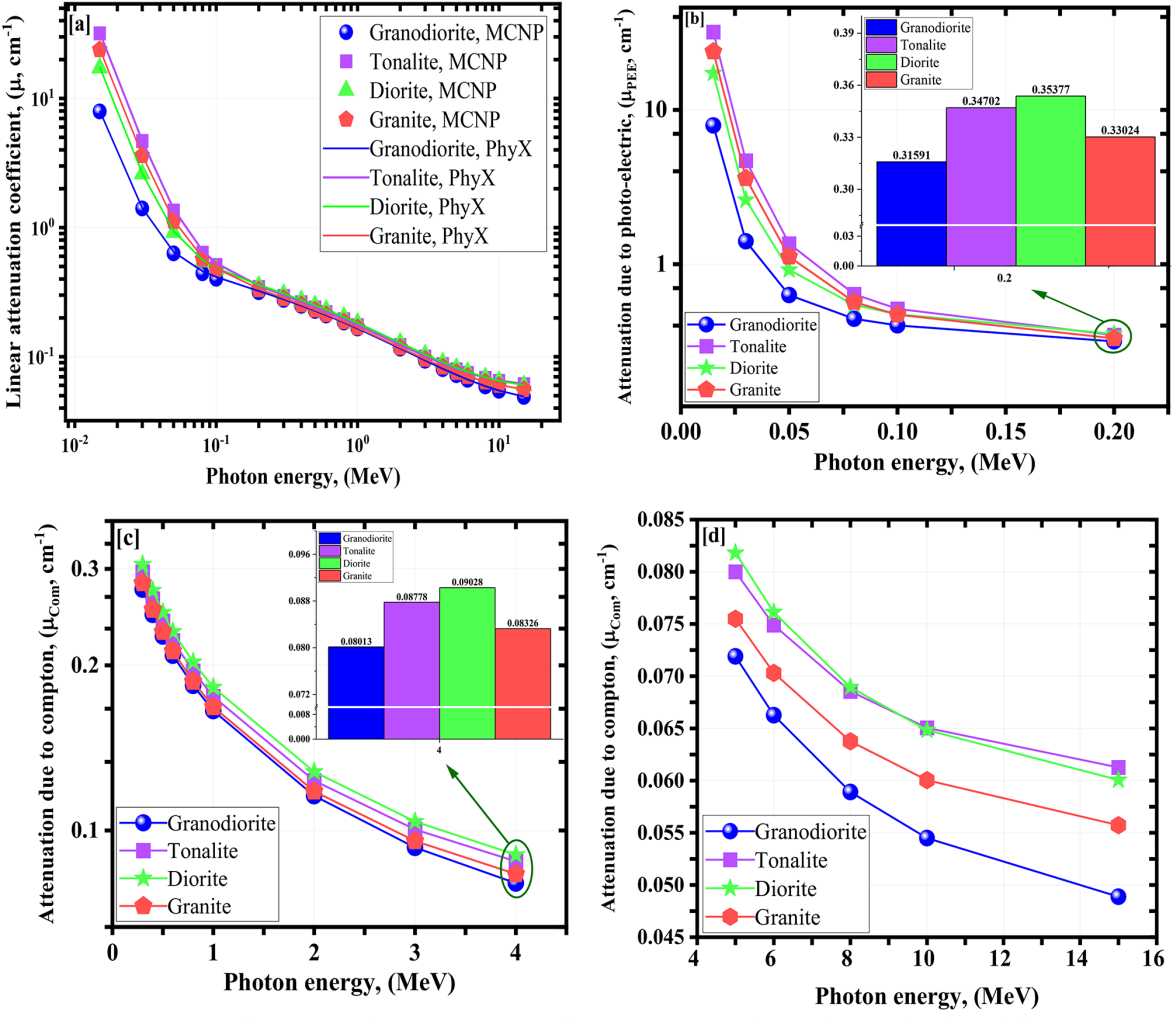
(**a–d**) Influence of gamma-ray energy on linear attenuation of (**a**) obtained from MCNP and PhyX, (**b**) photo electric, (**c**,**d**) compton scattering, for the investigated rock samples.

Before investigating the computed values of the various γ-rays’ shielding parameters, a comparison between the calculated (µ) values within a wide investigated energy range using both Phy-X/PSD software^51^ and the model created by MCNP^52,53^ has been performed (Table 3) using EDX analysis results (Fig. 2). The simulated µ values at all studied γ-rays’ energies are in excellent agreement with those calculated by the PhyX program, with a %Dev. that hasn’t reached 3.623% as seen in Fig. 5a. Figure 5b–d represent the µ’s behavior against the γ-energies. There were two trends of the µ’s decreasing as follows:

**Table 3:** The linear attenuation (*µ*), which was obtained using MCNP and PhyX for the rocks samples.

Energy, (MeV)	The linear (µ, cm^−1^)											
	Granodiorite			Tonalite			Diorite			Granite		
	PhyX	MCNP	Φ (%)	PhyX	MCNP	Φ (%)	PhyX	MCNP	φ (%)	PhyX	MCNP	Φ (%)
0.015	8.049	7.931	1.478	32.403	31.922	1.504	17.561	17.267	1.703	24.288	23.860	1.793
0.03	1.416	1.407	0.637	4.711	4.681	0.648	2.623	2.610	0.500	3.627	3.608	0.526
0.05	0.648	0.631	2.741	1.395	1.357	2.789	0.940	0.920	2.151	1.143	1.117	2.264
0.08	0.459	0.443	3.561	0.662	0.639	3.623	0.561	0.546	2.885	0.586	0.569	3.037
0.1	0.415	0.402	3.296	0.531	0.513	3.354	0.486	0.474	2.495	0.483	0.471	2.627
0.2	0.323	0.316	2.211	0.355	0.347	2.250	0.360	0.354	1.735	0.336	0.330	1.826
0.3	0.278	0.275	1.161	0.299	0.296	1.181	0.308	0.306	0.911	0.285	0.283	0.959
0.4	0.249	0.248	0.489	0.266	0.264	0.497	0.275	0.274	0.384	0.254	0.253	0.404
0.5	0.227	0.226	0.356	0.242	0.241	0.363	0.251	0.250	0.280	0.231	0.230	0.294
0.6	0.210	0.209	0.577	0.223	0.222	0.587	0.231	0.230	0.453	0.213	0.212	0.476
0.8	0.184	0.184	0.165	0.196	0.195	0.168	0.203	0.203	0.129	0.187	0.187	0.136
1	0.166	0.165	0.099	0.176	0.175	0.101	0.183	0.182	0.078	0.168	0.168	0.082
2	0.116	0.116	0.205	0.123	0.123	0.209	0.128	0.128	0.161	0.118	0.118	0.169
3	0.094	0.093	0.550	0.101	0.100	0.559	0.104	0.104	0.431	0.096	0.096	0.454
4	0.081	0.080	0.755	0.088	0.088	0.769	0.091	0.090	0.593	0.084	0.083	0.624
5	0.072	0.072	0.719	0.081	0.080	0.731	0.082	0.082	0.564	0.076	0.076	0.594
6	0.067	0.066	0.563	0.075	0.075	0.573	0.076	0.076	0.442	0.071	0.070	0.465
8	0.059	0.059	0.514	0.069	0.069	0.523	0.069	0.069	0.404	0.064	0.064	0.425
10	0.055	0.054	0.492	0.065	0.065	0.500	0.065	0.065	0.386	0.060	0.060	0.406
15	0.049	0.049	0.476	0.062	0.061	0.484	0.060	0.060	0.373	0.056	0.056	0.393

A. The trend due to the photo-electric effect^54–57^, which the cross-section (q) was proportional to the γe^− 4^. The increase of the applied γe values between γe ≤ 0.200 MeV causes a tough exponential decreasing tendency. The µ values were from 7.931 to 0.316 cm^−1^ for granodiorite, from 31.992 to 0.347 cm^−1^ for tonalite, from 17.267 to 0.354 cm^−1^ for diorite, and from 23.860 to 0.330 cm^−1^ for granite sample at γe ≤ 0.200.
B. The trend due to the Compton effect, in which the q was proportional to the γe^−1^. The upgrading in γe values was linked to a smooth decrease in the q with drops in the quantity of γ-electron interactions, followed by a smooth drop in the µ values. The µ values were from 0.275 to 0.049 cm^−1^ for granodiorite, from 0.296 to 0.061 cm^−1^ for tonalite, from 0.306 to 0.060 cm^−1^ for diorite, and from 0.283 to 0.056 cm^−1^ for granite sample at 0.0300 ≤ γe ≤≤ 15.

Table 4 lists the linear attenuation values for the rock samples and some other concretes (H-serpentine, I-limonite, B-magnetite, Ilmenite, Steel-scrap, and S-magnetite) and natural samples (marble, granite, and nano-alumina) at selected energies 0.5, 5, 10 MeV^26,63^. The investigated rocks samples were found higher than those compared.

**Table 4:** The linear attenuation for the rocks samples and some concretes and other natural samples.

Sample	Linear attenuation, cm^−1^			Reference
	5 MeV	10 MeV	0.5 MeV	
Granodiorite	0.072	0.054	0.226	This study
Tonalite	0.080	0.065	0.241	
Diorite	0.082	0.065	0.250	
Granite	0.076	0.060	0.230	
H-serpentine	0.030	0.025	0.087	^26^
I-limonite	0.030	0.026	0.086	
Bmagnetite	0.029	0.025	0.087	
Ilmenite	0.030	0.026	0.085	
Steel-scrap	0.032	0.027	0.085	
S-magnetite	0.031	0.028	0.085	
MD	0.077	0.063	0.229	^63^
GD	0.078	0.062	0.231	
NA	0.074	0.060	0.219	

**Fig. 6:**
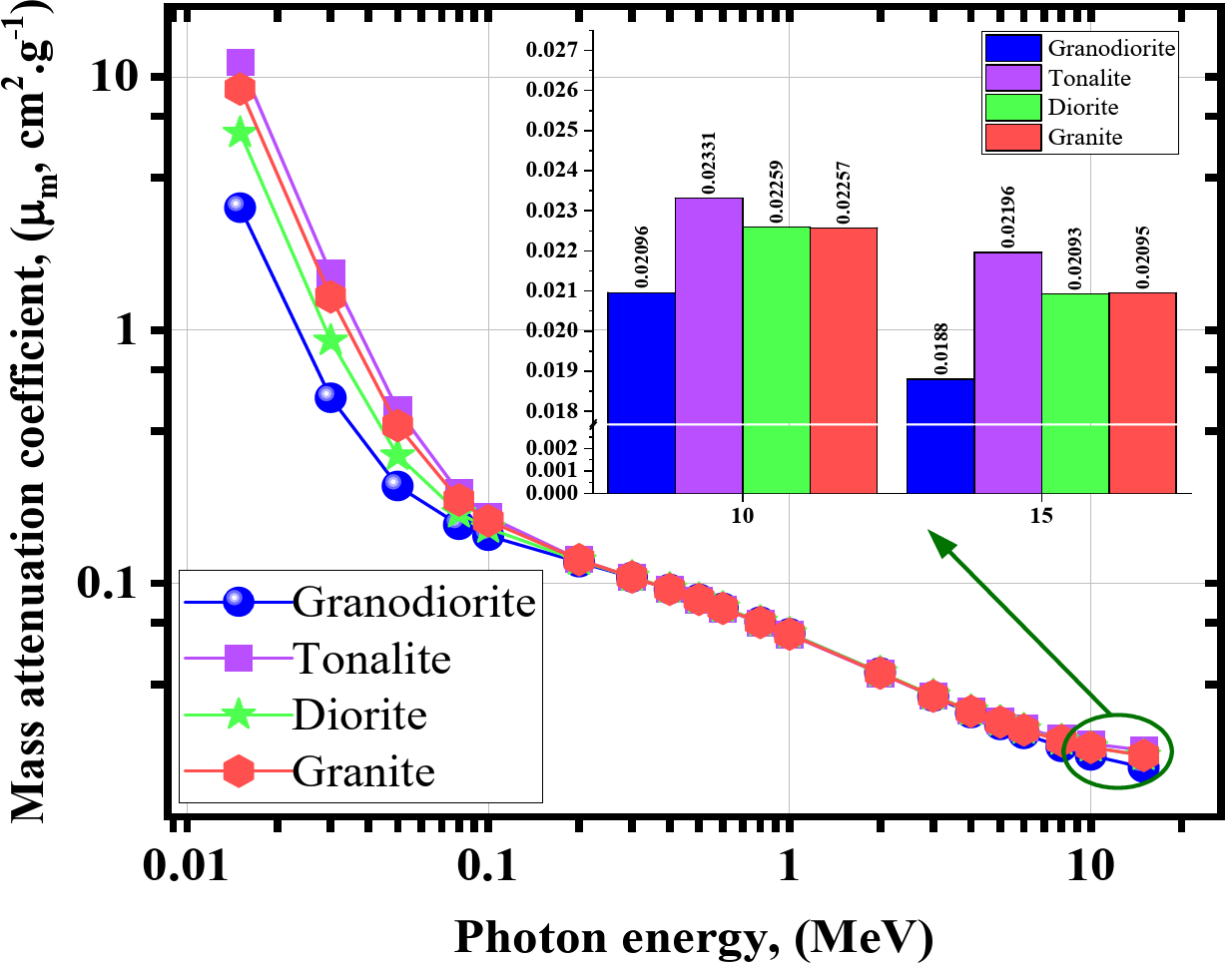
The mass attenuation coefficients (*µ*_*m*_) obtained from MCNP vs. the γ energy for the investigated rock samples.

Figure 6 represents the µ_c_’s behavior against the γ-energies. The µ_c_ values were from 3.050 to 0.019 cm^2^ g^− 1^ for granodiorite, from 11.442 to 0.022 cm^2^ g^− 1^ for tonalite, from 6.016 to 0.021 cm^2^ g^− 1^ for diorite, and from 8.970 to 0.021 cm^2^ g^− 1^ for granite sample at 0.015 ≤ γe ≤≤ 15.

**Fig. 7:**
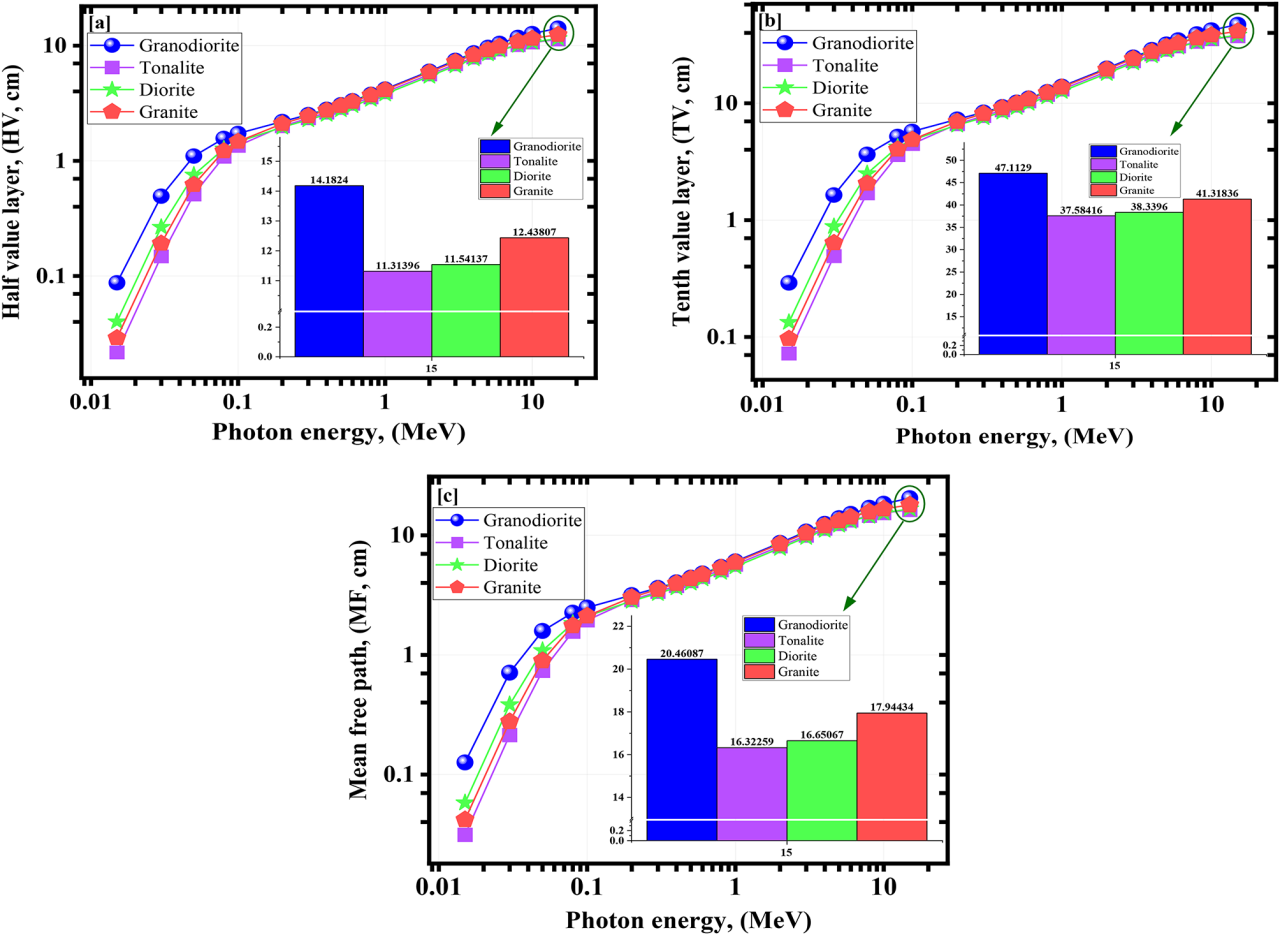
(**a**) Half value layer (HV), (**b**) Tenth value layer (TV), and (**c**) Mean free path (MF) for the investigated rock samples versus the γ-energy.

**Fig. 8:**
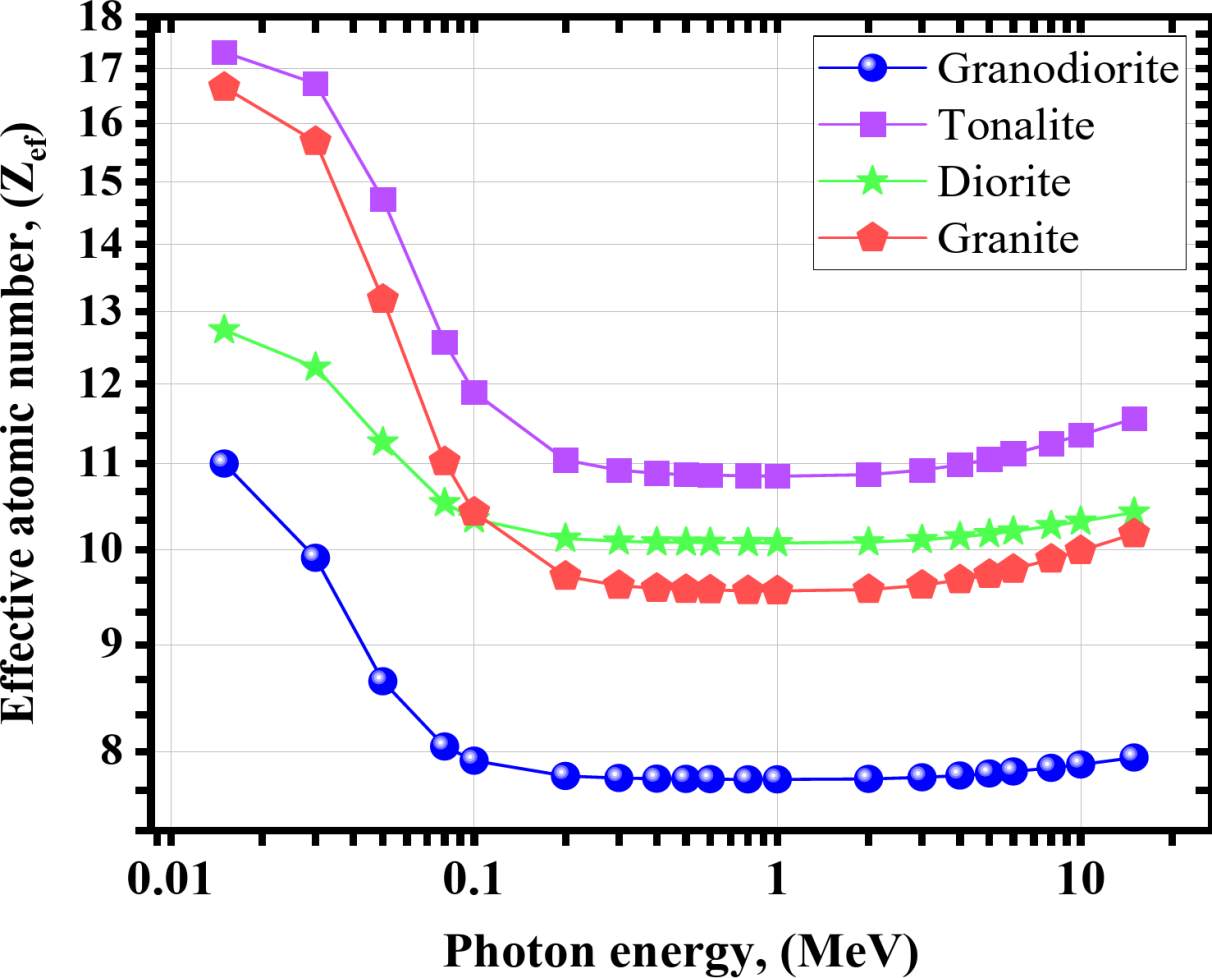
The effective atomic number (Z_ef_) for the investigated rock samples versus γ-energy.

The required shield thicknesses to attenuate are 50%, 90%, and about 67% of the incident photons, which are HV, TV, and MF, respectively^58–60^, are presented in Fig. 7a–c for the studied natural samples. The HV values were from 0.087 to 14.182 cm for granodiorite, from 0.022 to 11.314 cm for tonalite, from 0.040 to 11.539 cm for diorite, and from 0.029 to 12.438 cm for granite sample at 0.015 ≤ γe ≤ 15 as seen in Fig. 7a. The T_10/1_ values were from 0.290 to 47.113 cm for granodiorite, from 0.072 to 37.584 cm for tonalite, from 0.133 to 38.332 cm for diorite, and from 0.097 to 41.318 cm for granite sample at 0.015 ≤ γe ≤ 15 as seen in Fig. 7b. The MF parameter has the same trend as seen in Fig. 7c. Considering the effective atomic numbers (Z_eff_) and µ values computed for the studied rock samples within the energy range of interest γe ≤ 15, Fig. 8 depict the interrelation between the Z_eff_ shielding parameter and γ-energy.

Based on the results shown in the previous figures, Z_eff_ is known to be attributed to the γ-rays interaction modes with the attenuating medium; its value usually varies with the γ-energy^61–64^. As a consequence of this, higher values are observed at low energies as a result of the control of the γ-electric mechanism, which is significantly dependent on the Z of the shield constituents. On the other hand, for the current energy range that was studied, which ranged from 0.200 ≤ γe ≤ 15 MeV, the lowest values were observed throughout the majority of this range, with the exception of the onset, and they were almost independent of the γ-energy that was incident. That can be attributed to the dominancy of the Compton scattering mechanism at these energies^65–67^. Tonalite sample possesses the highest values, relatively, especially at the start of the studied energy range, as high-Z alloying elements such as FeO and TiO_2_. The Z_eff_ values were from 10.993 to 7.947 for granodiorite, from 17.312 to 11.553 for tonalite, from 12.740 to 10.417 cm for diorite, and from 16.655 to 10.174 for granite sample at γe ≤ 15.

**Fig. 9:**
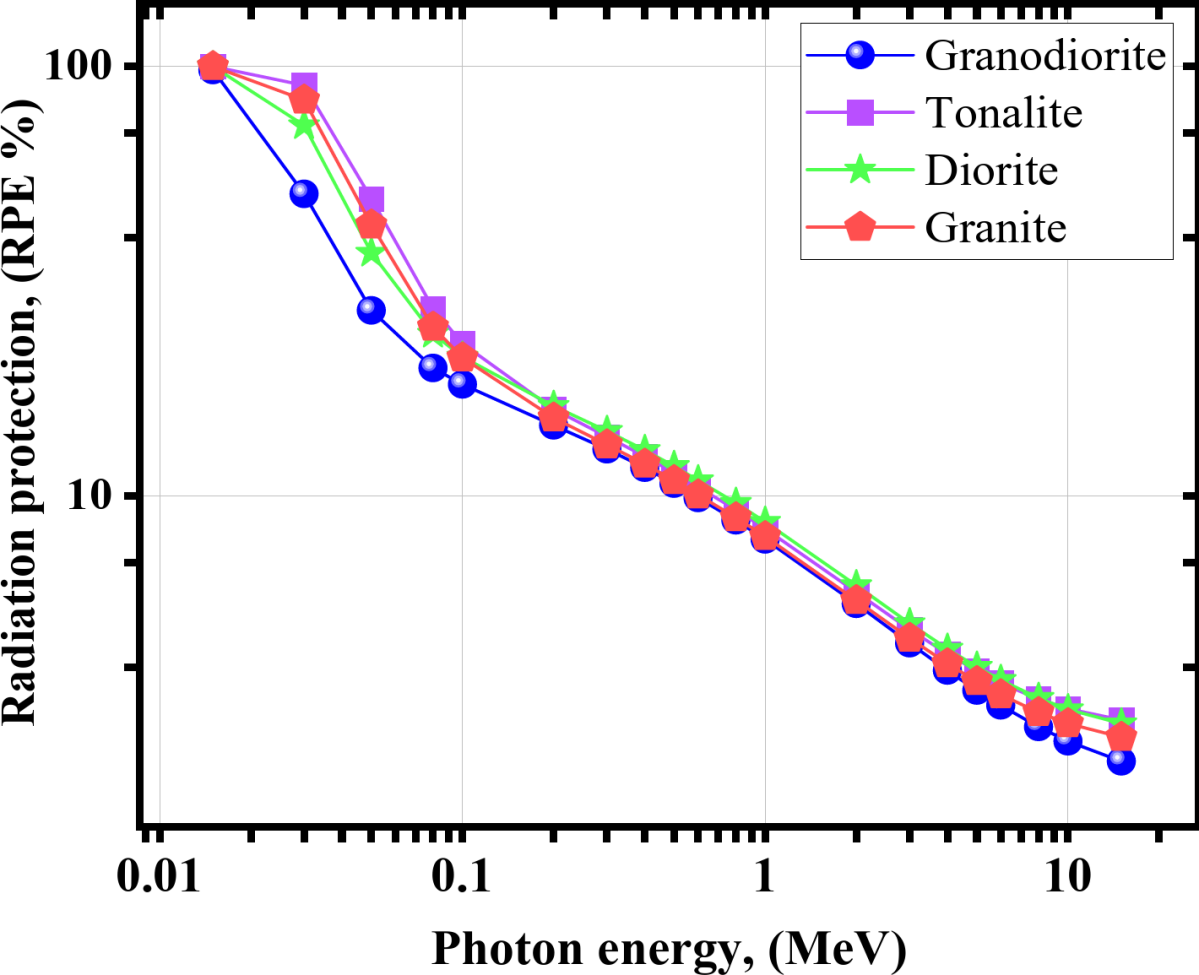
The RP for the investigated rock samples versus γ-energy.

As mentioned before, RP is an essential statistical parameter that should be taken into account when determining the level of attenuation that the shield could provide^63,68–71^. Figure 9 shows that the RP values are more than 18% at γe ≤ 0.1 MeV. When the incident γ-energy increases, the penetration power of the incident γs also increases, leading to a weighty decrease in the RP (%) levels. Therefore, at the start of the studied γ-energy range, the superiority of the tonalite sample over the other samples, thus, its γ-rays’ shielding efficiency, can be considered tangible and effective while dealing with low γ-rays’ radiation fields. The RP values dropped from about 18.191, 22.641, 21.092, and 20.985% at 0.100 MeV to only 2.414, 3.017, 2.958, and 2.748%, respectively, for all granodiorite, tonalite, diorite, and granite at γe = 15 MeV.

### Fast neutrons shielding assessment

**Fig. 10:**
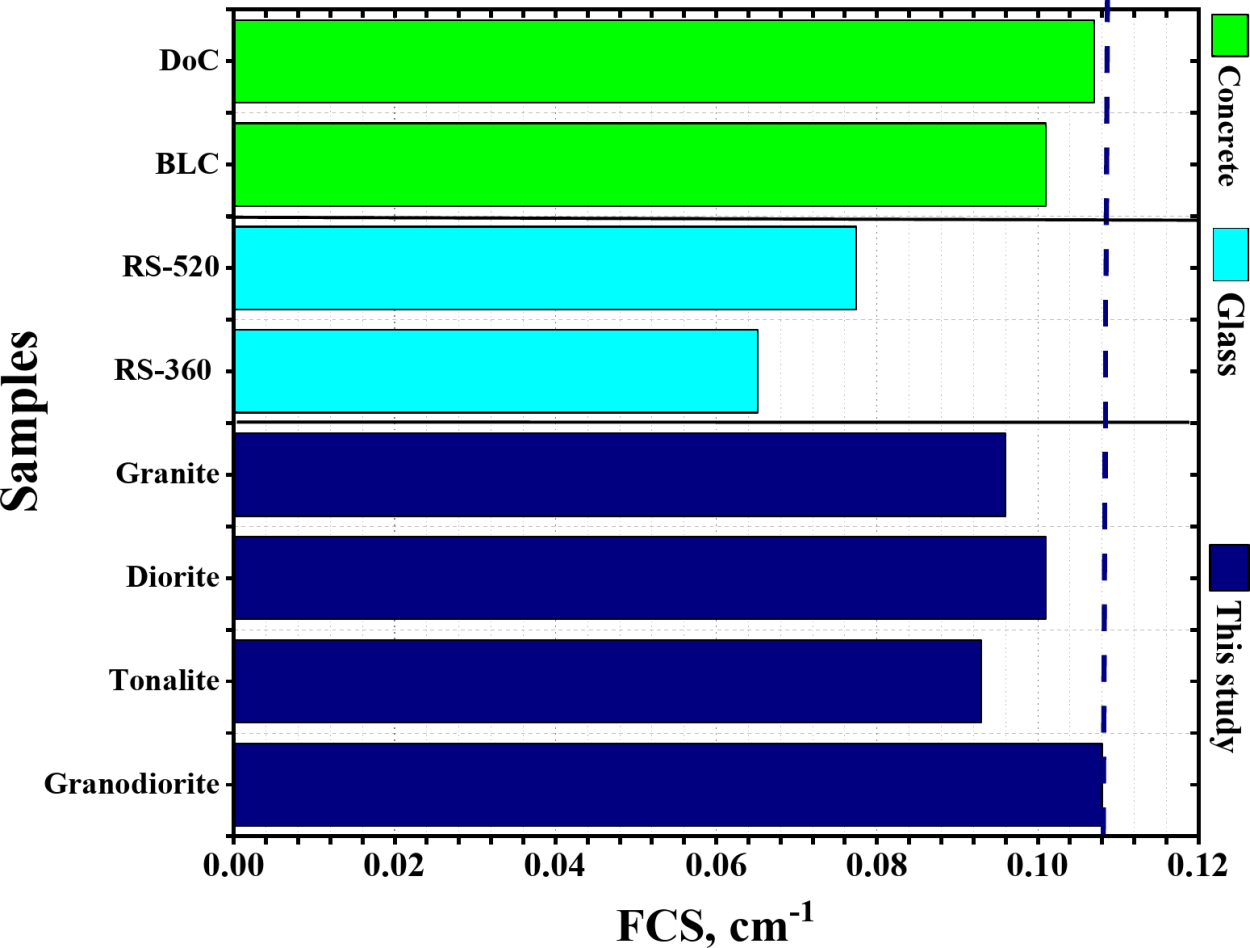
Comparison of the FCS for the investigated rock samples and commercial glass and concrete samples.

**Fig. 11:**
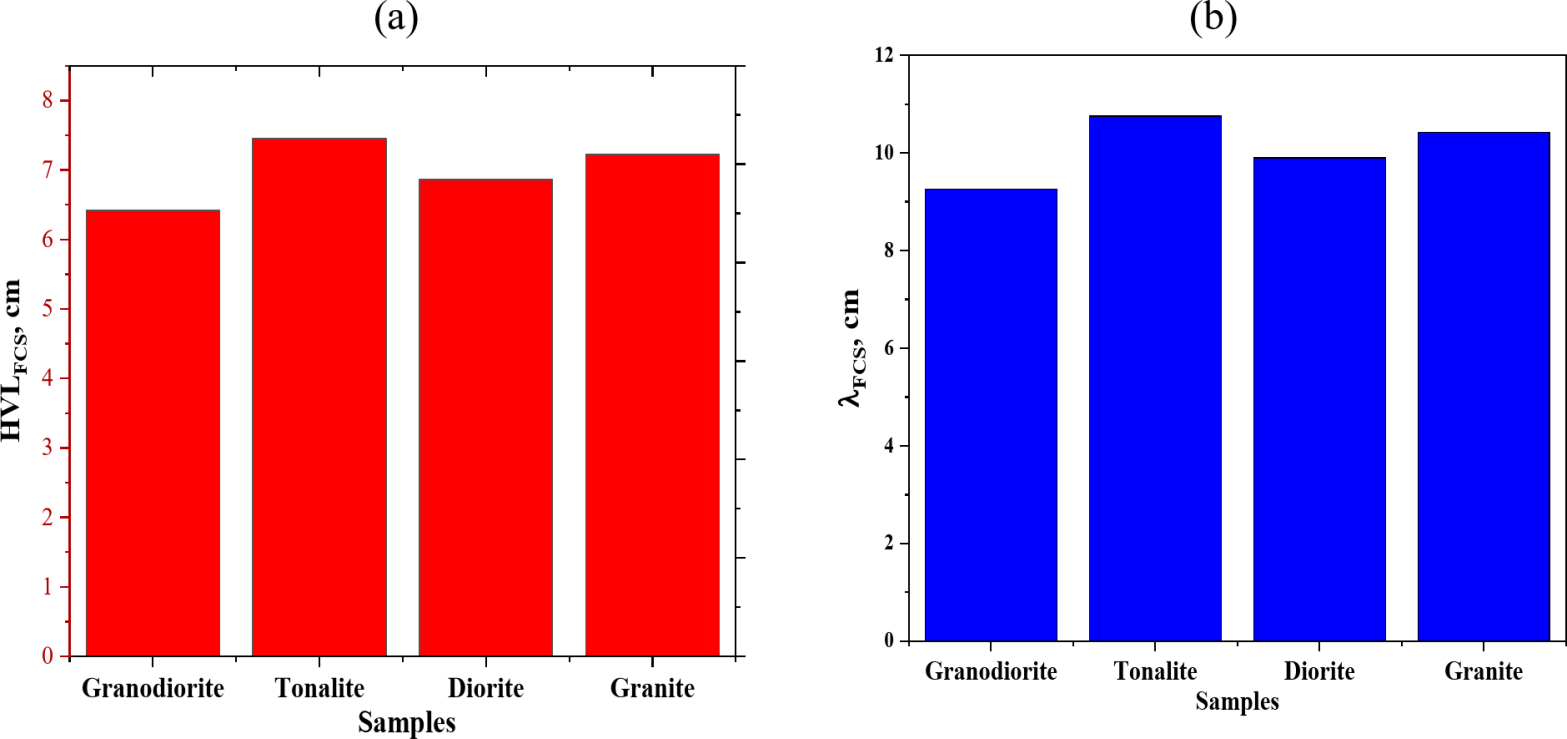
(**a**) The half value layer (HVL_FCS_), and (**b**) the relaxation length (λ_FCS_) for the investigated rock samples.

The neutron shielding of a material is one of the parameters that determines the extent of the material’s ability to block fast neutrons, by calculating the FCS, HVL_FCS_, and λ_FCS_. Figure 10 represents a comparison of the FCS for the investigated rock samples and some commercial glass and concrete samples. Table 5 lists the chemical composition of the glass and concrete samples which compared with the rock samples. Figure 11 represents the half value layer (HVL_FCS_), and the relaxation length (λ_FCS_) for the investigated rock samples. The granodiorite samples were in first place with average Σ_R_ equals 0.108 cm^− 1^ and corresponding average HVL_FCS_ and λ_FCS_ equal 6.418 and 9.259 cm, respectively. In contrast, tonalite has the most negligible Σ_R_ value (0.093 cm^− 1^) and the most significant thicknesses for both HVL and λ, equal to 7.453 and 10.753 cm, respectively. Possessing the high content of low-Z elements like carbon and oxygen (0.228, 0.680 wt.%) can be considered the reason for putting granodiorite sample in the lead regarding attenuating fast neutrons that can be achieved by increasing neutrons and samples interactions depending mainly on the inelastic scattering mechanism.

**Table 5:** The chemical composition of the glass and concrete samples which compared with the rock samples.

Element	Compounds composition (wt.%)			
	BLC	DOC	RS-360	RS-520
H	0.0096	0.012402	–	–
B	–	–	–	–
C	0.0009	0.052911	–	–
O	0.3217	0.523092	0.1699	0.1158
Na	0.0005	0.001716	0.0034	0.0052
Mg	0.0081	0.045964	–	–
Al	0.0072	0.011324	–	–
Si	0.065738	0.12849	0.0925	0.0429
S	0.0132	0.003213	–	–
K	0.0021	0.002004	0.0515	0.8317
Ca	0.084463	0.210201	–	–
Ti	0.0006	0.000665	–	–
Cr	0.0011	–	–	–
Mn	0.005	–	–	–
Fe	0.1247	0.008018	–	–
Ba	0.355	–	–	–
Pb	–	–	0.6827	0.0044
Density (g cm^− 3^)	3.044	2.4310	3.600	5.200

## Conclusions

This study investigates four natural rock samples (diorite, granodiorite, tonalite, and granite) are which investigated about their radiation attenuation. Energy dispersive X-ray spectroscopy (EDX) was used to examines the microstructural and localized area elemental analyses of the four rock samples. Based on the intensive experimental and analytical investigation conducted through this study, the following conclusions were drawn.

- The superiority of tonalite samples in shielding against energetic ionizing γ-photons was appreciated for low-energy γ-rays, indicating the feasibility of using these samples as a shield against X-ray radiation fields.
- The superiority of the diorite sample in shielding against energetic ionizing γ-photons was appreciated for higher-energy γ-rays.
- Considering attenuation capabilities against fast neutrons, granodiorite sample was the best but with a slight degree of superiority above the other studied rock samples.

## Data Availability

All data generated or analyzed during this study are included in this published article.

## References

[1] Phase, B. *Health Risks from Exposure to Low Levels of Ionizing Radiation* (The British Institute of Radiology, 2006).

[2] Nabil, I. M., El-Samrah, M. G., Sayed, A. F. E., Shazly, A. & Omar, A. Radionuclides distribution and radiation hazards assessment of black sand separation plant’s minerals: a case study. *Sci. Rep.* **14**, 5241. 10.1038/s41598-024-55633-1 (2024). 38438490 10.1038/s41598-024-55633-1 PMC11319471

[3] El-Rehim, A. F. A., Zahran, H. Y., Yahia, I. S., Makhlouf, S. A. & Shaaban, K. S. Radiation, crystallization, and physical properties of cadmium borate glasses. *Silicon* **13**, 2289–2307. 10.1007/s12633-020-00798-3 (2020).

[4] Kaewkhao, J. et al. Monte Carlo design and experiments on the neutron shielding performances of B2 O3–ZnO–Bi2 O3 glass system. *Glass Phys. Chem.* **43**, 560–563 (2017).

[5] Al-Buriahi, M. S. et al. Dense and environment friendly bismuth barium telluroborate glasses for nuclear protection applications. *Prog. Nucl. Energy* **137**, 103763 (2021).

[6] Shahzad, K. et al. Views on radiation shielding efficiency of polymeric composites/nanocomposites and multi-layered materials: current state and advancements. *Radiation* **3**, 1–20 (2023).

[7] Al-Saleh, W. M., Almutairi, H. M., Sayyed, M. & Elsafi, M. Multilayer radiation shielding system with advanced composites containing heavy metal oxide nanoparticles: a free-lead solution. *Sci. Rep.* **13**, 18429 (2023). 37891224 10.1038/s41598-023-45621-2 PMC10611706

[8] Al-Buriahi, M., Gaikwad, D., Hegazy, H., Sriwunkum, C. & Algarni, H. Newly developed glasses containing Si/Cd/Li/Gd and their high performance for radiation applications: role of Er2 O3. *J. Mater. Sci. Mater. Electron.* **32**, 9440–9451 (2021).

[9] Wang, J. Focuses of material science development in recent years. *Sci. China Technol. Sci.* **54**, 1645–1648. 10.1007/s11431-011-4383-3 (2011).

[10] Saleh, A., El-Feky, M. G., Hafiz, M. S. & Kawady, N. A. Experimental and theoretical investigation on physical, structure and protection features of TeO2–B2O3 glass doped with PbO in terms of gamma, neutron, proton and alpha particles. *Radiat. Phys. Chem.* **202**, 110586. 10.1016/j.radphyschem.2022.110586 (2023).

[11] Bonewitz, R. *Rocks and Minerals* (DK Publishing, 2012).

[12] Abou Hussein, E. Gamma rays interactions on optical, FTIR and ESR spectra of alkaline earth binary borate glasses. *Arab. J. Nucl. Sci. Appl.* **53**, 1–18 (2020).

[13] El-Nahal, M. A. et al. Understanding the effect of introducing micro- and nanoparticle bismuth oxide (Bi(2)O(3)) on the gamma ray shielding performance of novel concrete. *Materials (Basel)* **14**, 6487. 10.3390/ma14216487 (2021). 10.3390/ma14216487 PMC8585322 34772013

[14] Montgomery, C. W. & Szablewski, G. S. *Environmental Geology* (McGraw-Hill, 2006).

[15] Eslamian, S. *Handbook of Engineering Hydrology: Fundamentals and Applications* (CRC, 2014).

[16] Elsafi, M. et al. The potentials of Egyptian and Indian granites for protection of ionizing radiation. *Materials (Basel)* **14**, 3928. 10.3390/ma14143928 (2021). 34300846 10.3390/ma14143928 PMC8304081

[17] Alsaif, N. A. M. et al. The impact of TiO2 on physical, optical characteristics and shielding qualities against γ-ray features of titanium bismo-borate glasses. *Opt. Quant. Electron.* **56**, 816. 10.1007/s11082-024-06702-2 (2024).

[18] Alharshan, G. A. et al. CeO2 additive to bismo-borate glasses: synthesis, structure, physical characteristics, and radiation protection competence. *J. Mater. Sci. Mater. Electron.* **35**, 862. 10.1007/s10854-024-12610-8 (2024).

[19] Günoğlu, K., Akkurt, I. & Sayyed, M. Radiation shielding properties of some igneous rocks in Isparta province at different gamma energies: experimental and theoretical study. *J. Radiat. Res. Appl. Sci.* **17**, 100796 (2024).

[20] Masoud, M. A. et al. Deep insights into the radiation shielding features of heavy minerals in their native status: implications for their physical, mineralogical, geochemical, and morphological properties. *Sustainability* **14**, 16225 (2022).

[21] Rashwan, M. A. et al. Physico-mechanical properties and shielding efficiency in relation to mineralogical and geochemical compositions of Um had granitoid, Central Eastern Desert, Egypt. *Front. Earth Sci.* **11**, 1228489 (2023).

[22] Sekhar, K. C. et al. Synthesis, optical, structural, and radiation transmission properties of PbO/Bi2O3/B2O3/Fe2O3 glasses: an experimental and in silico study. *Opt. Mater.* **117**, 111173 (2021).

[23] Alsaif, N. A. M. et al. Influence of WO3 replacement for CaO on physical, optical, and γ-ray protection properties of borotellurite glasses: a comparative study. *Ceram. Int.*10.1016/j.ceramint.2024.06.076 (2024).

[24] Almutairi, H. M., Al-Saleh, W. M., Abualsayed, M. I. & Elsafi, M. Effect of cerium (IV) oxide particle size on polydimethylsiloxane polymer to form flexible materials against ionizing radiation. *Polymers* **15**, 2883 (2023). 37447530 10.3390/polym15132883 PMC10346377

[25] Al-Ghamdi, H. et al. Sustainable wastewater sludge@bimetallic cadmium-MOFs and nano-copper oxide as a promising shielding composite for gamma rays: experimental and simulation investigations. *Mater. Sci. Eng. B* **308**, 117609. 10.1016/j.mseb.2024.117609 (2024).

[26] El-Samrah, M., Zamora, M. A., Novog, D. & Chidiac, S. Radiation shielding properties of modified concrete mixes and their suitability in dry storage cask. *Prog. Nucl. Energy* **148**, 104195 (2022).

[27] Abouhaswa, A. et al. The impact of B2O3/Al2O3 substitution on physical properties and γ-ray shielding competence of aluminum-borate glasses: comparative study. *J. Mater. Sci. Mater. Electron.* **35**, 1–13. 10.1007/s10854-024-12629-x (2024).

[28] Hussein, E. A. & Madbouly, A. Chemical and radiation shielding effectiveness of some heavy metal oxide glasses for immobilizing radioactive wastes. *J. Aust. Ceram. Soc.* **60**, 127–142 (2024).

[29] Al-Buriahi, M. Radiation shielding performance of a borate-based glass system doped with bismuth oxide. *Radiat. Phys. Chem.* **207**, 110875 (2023).

[30] Aloraini, D. A., Elsafi, M., Almuqrin, A. H. & Sayyed, M. Coincidence summing factor calculation for volumetric γ-ray sources using Geant4 simulation. *Sci. Technol. Nucl. Install.* 5718920 (2022).

[31] Aygun, B. et al. Development of novel composite materials containing rice bran wax and waste polyethylene for neutron shielding applications. *Prog. Nucl. Energy* **174**, 105262 (2024).

[32] Al-Ghamdi, H. et al. Strontium oxide-reinforced borotellurite glasses: synthesis, structure, and optical characteristics and γ-ray and neutron attenuation capability. *J. Electron. Mater.*10.1007/s11664-024-11201-x (2024).

[33] Abou Hussein, E. M. & Gafar, S. M. Effect of gamma rays on Zn/Cu doped strontium borate glass system for dosimetric applications. *Radiochim. Acta*. **110**, 913–923 (2022).

[34] Nabil, I. M. et al. Evaluation of the effect of low additive V2O5/ZnO on the structural, physical, and radiation attenuation performance of borate glasses. *J. Mater. Sci. Mater. Electron.* **35**, 1532. 10.1007/s10854-024-13244-6 (2024).

[35] Al-Ghamdi, H., Alsaif, N. A. M., Alfryyan, N., Rammah, Y. S. & Nabil, I. M. Investigation of gamma-ray and neutron protection competence of oxyfluoride aluminosilicate glasses reinforced with TbF3: comparative study. *Radiat. Phys. Chem.* 112105. 10.1016/j.radphyschem.2024.112105 (2024).

[36] Aygün, B. Neutron and gamma radiation shielding properties of high-temperature-resistant heavy concretes including chromite and wolframite. *J. Radiat. Res. Appl. Sci.* **12**, 352–359 (2019).

[37] Didi, A., Dadouch, A., Bencheikh, M. & Jai, O. Monte Carlo simulation of thermal neutron flux of americium–beryllium source used in neutron activation analysis. *Mosc. Univ. Phys. Bull.* **72**, 460–464 (2017).

[38] El-Samrah, M., El-Mohandes, A., El-Khayatt, A. & Chidiac, S. MRCsC: a user-friendly software for predicting shielding effectiveness against fast neutrons. *Radiat. Phys. Chem.* **182**, 109356 (2021).

[39] Didi, A., Dadouch, A. & Jai, O. Modelisation and distribution of neutron flux in radium–beryllium source (226 Ra–Be). *Mosc. Univ. Phys. Bull.* **72**, 465–469 (2017).

[40] Al-Saleh, W. M., Elsafi, M., Almutairi, H. M., Nabil, I. M. & El-Nahal M. A. A comprehensive study of the shielding ability from ionizing radiation of different mortars using iron filings and bismuth oxide. *Sci. Rep.* **14**, 10014. 10.1038/s41598-024-60188-2 (2024). 38693293 10.1038/s41598-024-60188-2 PMC11063177

[41] Nabil, I. M., Ebaid, Y. Y. & El-Mongy, S. A. Natural radionuclides quantification and radiation hazard evaluation of phosphate fertilizers industry: a case study. *Phys. Part. Nucl. Lett.* **19**, 272–281. 10.1134/S1547477122030177 (2022).

[42] Nabil, I. M., El-Kourghly, K. M. & El Sayed, A. F. A semi-empirical method for efficiency calibration of an HPGe detector against different sample densities. *Appl. Radiat. Isot.* **200**, 110946. 10.1016/j.apradiso.2023.110946 (2023). 37523863 10.1016/j.apradiso.2023.110946

[43] Zayed, A. et al. Physical, mechanical, and radiation attenuation properties of serpentine concrete containing boric acid. *Constr. Build. Mater.* **272**, 121641 (2021).

[44] Masoud, M. A. et al. Radiation attenuation assessment of serpentinite rocks from a geological perspective. *Toxics* **10**, 697 (2022). 36422904 10.3390/toxics10110697 PMC9698313

[45] Alharshan, G. A. et al. Effect of lanthanum oxide on the radiation-shielding, dielectric, and physical properties of lithium zinc phosphate glasses. *Radiat. Phys. Chem.* **224**, 112053. 10.1016/j.radphyschem.2024.112053 (2024).

[46] Alfryyan, N. et al. SiO2/Ag2O substitution of borosilicate glasses: preparation, structure, physical features and γ-ray protection capability. *Silicon*. 10.1007/s12633-024-03059-9 (2024).

[47] Nabil, I. M. et al. Influence of low copper oxide additives on B2O3-Li2O-Na2O-CaO-SrO-As2O3 glasses: a physical, structural, and radiological study. *J. Mater. Sci. Mater. Electron.* **35**, 1329. 10.1007/s10854-024-12891-z (2024).

[48] Team, M. C. *MCNP–a General Monte Carlo N-Particle Transport Code (X-5 Monte Carlo Team, Version 5). Vol. I: Overview and Theory* (Los Alamos National Laboratory, LA-UR-03-1987, 2003).

[49] Briesmeister, J. F. MCNPTM-A general Monte Carlo N-particle transport code. *Version 4 C, LA-13709-M, Los Alamos National Laboratory* 2 (2000).

[50] Alsaif, N. A. M. et al. Optical and gamma-ray attenuation of cobalt and lanthanum-doped sodium zinc lead borate glass. *J. Mater. Sci. Mater. Electron.* **35**, 1458. 10.1007/s10854-024-13168-1 (2024).

[51] Şakar, E. et al. -X/PSD: development of a user friendly online software for calculation of parameters relevant to radiation shielding and dosimetry. *Radiat. Phys. Chem.* **166**, 108496 (2020).

[52] Alsaif, N. A. M. et al. Linear/non-linear optical properties, γ-ray and neutron shielding competence of borosilicate glasses reinforced with Ag2O. *J. Mater. Sci. Mater. Electron.* **35**, 1604. 10.1007/s10854-024-13344-3 (2024).

[53] Team, M. C. MCNP–a general Monte Carlo N-particle transport code, version 5. *Book MCNP-A General Monte Carlo N-Particle Transport Code Version* 5 (2003).

[54] Al-Saleh, W. M., Elsafi, M., Almutairi, H. M., Nabil, I. M. & El-Nahal, M. A comprehensive study of the shielding ability from ionizing radiation of different mortars using iron filings and bismuth oxide. *Sci. Rep.* **14**, 10014 (2024). 38693293 10.1038/s41598-024-60188-2 PMC11063177

[55] Kassem, S. M. et al. Optical and radiation shielding properties of PVC/BiVO4 nanocomposite. *Sci. Rep.* **13**, 10964 (2023). 37415084 10.1038/s41598-023-37692-y PMC10326021

[56] Sayyed, M. et al. Exploring gamma radiation shielding: the role of BaO in borosilicate glasses. *Silicon* **1**, 1–10 (2024).

[57] Thabit, H. A. et al. Optical, thermal and gamma ray attenuation characteristics of tungsten oxide modified: B2O3–SrCO3–TeO2–ZnO glass series. *Nucl. Eng. Technol.* **56**, 247–256 (2024).

[58] Lamarsh, J. R. & Baratta, A. J. *Introduction to Nuclear Engineering*, Vol. 3 (Prentice Hall, 2001).

[59] Abou Hussein, E., Shaban, S., Rammah, Y., Misbah, M. H. & Marzouk, M. Newly developed CeO2 and Gd2O3-reinforced borosilicate glasses from municipal waste ash and their optical, structural, and gamma-ray shielding properties. *Sci. Rep.* **14**, 13673 (2024). 38871825 10.1038/s41598-024-63207-4 PMC11176321

[60] Abou Hussein, E., Barakat, M. A. & Marzouk, M. The role of CeO2 insertion and gamma radiation in optoelectronic and ultrasonic transducer applications of PbO-B2O3 glasses. *J. Non-cryst. Solids* **628**, 122861 (2024).

[61] Alresheedi, M. T. et al. Assessment of silicone rubber/lead oxide composites enriched with Bi2O3, WO3, BaO, and SnO2 nanoparticles for radiation shielding applications. *Polymers* **15**, 2160 (2023). 37177306 10.3390/polym15092160 PMC10180752

[62] Taylor, M., Smith, R., Dossing, F. & Franich, R. Robust calculation of effective atomic numbers: the auto-zeff software. *Med. Phys.* **39**, 1769–1778 (2012). 22482600 10.1118/1.3689810

[63] Mahmoud, A. A. et al. Influence of sustainable waste granite, marble and nano-alumina additives on ordinary concretes: a physical, structural, and radiological study. *Sci. Rep.* **14**, 22011 (2024). 39317712 10.1038/s41598-024-72222-4 PMC11422509

[64] Sallam, O. I., Rammah, Y. S., Nabil, I. M. & El-Seidy, A. M. A. enhanced optical and structural traits of irradiated lead borate glasses via Ce3 + and Dy3 + ions with studying Radiation shielding performance. *Sci. Rep.* **14**, 24478. 10.1038/s41598-024-73892-w (2024). 39424847 10.1038/s41598-024-73892-w PMC11489820

[65] Abou Hussein, E., Abd Elaziz, T. & El-Alaily, N. Effect of gamma radiation on some optical and electrical properties of lithium bismuth silicate glasses. *J. Mater. Sci. Mater. Electron.* **30**, 12054–12064 (2019).

[66] Abou Hussein, E., Maksoud, M. A., Fahim, R. A. & Awed, A. Unveiling the gamma irradiation effects on linear and nonlinear optical properties of CeO2–Na2O–SrO–B2O3 glass. *Opt. Mater.* **114**, 111007 (2021).

[67] Abou Hussein, E. & Madbouly, A. El Alaily, N. Gamma ray interaction of optical, chemical, physical behavior of bismuth silicate glasses and their radiation shielding proficiency using Phy-X/PSD program. *J. Non-cryst. Solids* **570**, 121021 (2021).

[68] Abdel-Gawad, E. H., Sayyed, M., Hanafy, T. A. & Elsafi, M. Experimental investigation of radiation shielding competence of B2O3-Na2O-Al2O3-BaO-CaO glass system. *Sci. Rep.* **14**, 14891 (2024). 38937501 10.1038/s41598-024-63329-9 PMC11211421

[69] Fathy, I. N. et al. Enhancing mechanical properties and radiation shielding of high-strength concrete with bulk lead oxide and granodiorite. *Nucl. Eng. Des.* **429**, 113626. 10.1016/j.nucengdes.2024.113626 (2024).

[70] Nabil, I. M. et al. Lithium magnesium borosilicate glass: the impact of alternate doping with nano copper oxide and nano hematite on its structural, optical, and nuclear radiation shielding characteristics. *J. Mater. Sci. Mater. Electron.* **35**, 826 (2024).

[71] Almuqrin, A. H., Sayyed, M. & Elsafi, M. A sustainable improvement of mortar by incorporation of marble dust and Zr2O3-NPs for radiation shielding applications. *Radiat. Phys. Chem.* **212**, 111111 (2023).

