# Author Correction: Unveiling the radiation shielding efficacy of diorite, granodiorite, tonalite, and granite: experimental and simulation study

**DOI:** 10.1038/s41598-025-93450-2

**Published:** 2025-03-19

**Authors:** M. Elsafi, M. A. El-Nahal, M. K. Alawy, Islam M. Nabil

**Affiliations:** 1 Physics Department, Faculty of Science, Alexandria University, Alexandria, 21511 Egypt; 2 Department of Environmental Studies, Institute of Graduate Studies and Research, Alexandria University, Alexandria, 21511 Egypt; 3 MEU Research Unit, Middle East University, Amman, Jordan; 4 Geology Department, Faculty of Science, Alexandria University, Alexandria, 21511 Egypt; 5 Physics Department, Faculty of Science, Fayoum University, Fayoum, Egypt

Correction to: *Scientific Reports* 10.1038/s41598-024-82081-8, published online 04 January 2025

In the original version of this Article, the descriptive names ‘Granite’ and ‘Granodiorite’ were incorrectly displayed.

The original Figure 1 and its accompanying legends appear below.

**Fig. 1:**
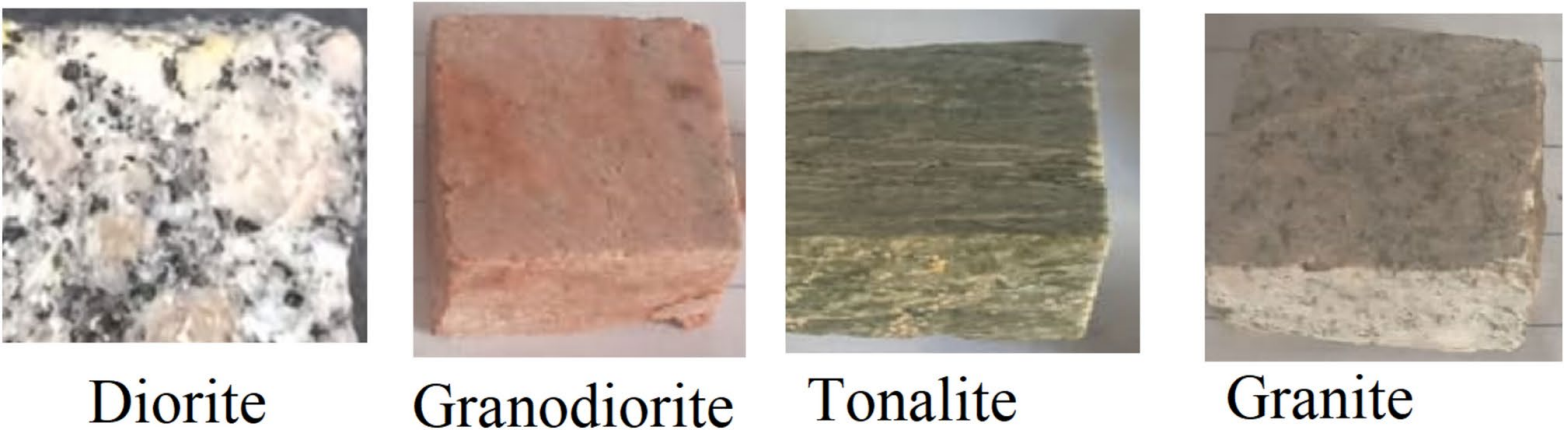
Samples images for investigated rock samples.

The original Article has been corrected.

